# The development of the WHO Labour Care Guide: an international survey of maternity care providers

**DOI:** 10.1186/s12978-021-01074-2

**Published:** 2021-03-22

**Authors:** Veronica Pingray, Mercedes Bonet, Mabel Berrueta, Agustina Mazzoni, María Belizán, Netanya Keil, Joshua Vogel, Fernando Althabe, Olufemi T. Oladapo

**Affiliations:** 1 Department of Mother and Child Health Research, Institute for Clinical Effectiveness and Health Policy (IECS-CONICET), Buenos Aires, Argentina; 2 UNDP/UNFPA/UNICEF/WHO/World Bank Special Programme of Research, Development and Research Training in Human Reproduction (HRP), Department of Sexual and Reproductive Health and Research, World Health Organization, Geneva, Switzerland; 3 New York University, Abu Dhabi, United Arab Emirates; 4 Maternal, Child and Adolescent Health Program, Burnet Institute, Melbourne, Australia

**Keywords:** Childbirth, Intrapartum care, Labour, Partograph, WHO labour care guide

## Abstract

**Background:**

The partograph is the most commonly used labour monitoring tool in the world. However, it has been used incorrectly or inconsistently in many settings. In 2018, a WHO expert group reviewed and revised the design of the partograph in light of emerging evidence, and they developed the first version of the Labour Care Guide (LCG). The objective of this study was to explore opinions of skilled health personnel on the first version of the WHO Labour Care Guide.

**Methods:**

Skilled health personnel (including obstetricians, midwives and general practitioners) of any gender from Africa, Asia, Europe and Latin America were identified through a large global research network. Country coordinators from the network invited 5 to 10 mid-level and senior skilled health personnel who had worked in labour wards anytime in the last 5 years. A self-administered, anonymous, structured, online questionnaire including closed and open-ended questions was designed to assess the clarity, relevance, appropriateness of the frequency of recording, and the completeness of the sections and variables on the LCG.

**Results:**

A total of 110 participants from 23 countries completed the survey between December 2018 and January 2019. Variables included in the LCG were generally considered clear, relevant and to have been recorded at the appropriate frequency. Most sections of the LCG were considered complete. Participants agreed or strongly agreed with the overall design, structure of the LCG, and the usefulness of reference thresholds to trigger further assessment and actions. They also agreed that LCG could potentially have a positive impact on clinical decision-making and respectful maternity care. Participants disagreed with the value of some variables, including coping, urine, and neonatal status.

**Conclusions:**

Future end-users of WHO Labour Care Guide considered the variables to be clear, relevant and appropriate, and, with minor improvements, to have the potential to positively impact clinical decision-making and respectful maternity care.

**Supplementary Information:**

The online version contains supplementary material available at 10.1186/s12978-021-01074-2.

## Resumen

**Antecedentes:**

El partograma es la herramienta para el monitoreo del trabajo de parto más utilizada a nivel mundial. Sin embargo, en muchos entornos se utiliza de manera incorrecta o inconsistente. En 2018, un grupo de expertos de la OMS evaluó y modificó el diseño del partograma teniendo en cuenta la nueva evidencia científica, y elaboró la primera versión de la Guía para la Atención del Trabajo de Parto. El objetivo de este estudio fue explorar las perspectivas y opiniones de los profesionales de la salud sobre la primera versión de la Guía para la Atención del Trabajo de Parto.

**Métodos:**

Se identificó a profesionales de salud de servicios de maternidad (incluidos obstetras, parteras profesionales y médicos generalistas) de cualquier género de África, Asia, Europa y América Latina a través de una red de investigación internacional. Los coordinadores de países de la red invitaron entre 5 y 10 profesionales con nivel de experiencia media o avanzada que hubieran trabajado en servicios de maternidad en los últimos 5 años. Se diseñó un cuestionario en línea, autoadministrado, anónimo y estructurado que incluía preguntas cerradas y abiertas para evaluar la claridad, la pertinencia, la adecuación de la frecuencia de registro y la exhaustividad de las secciones y variables de la Guía para la Atención del Trabajo de Parto.

**Resultados:**

Un total de 110 participantes de 23 países completaron la encuesta entre diciembre de 2018 y enero de 2019. Las variables incluidas en la Guía para la Atención del Trabajo de Parto se consideraron en general claras, pertinentes y con una frecuencia de registro apropiada. La mayoría de las secciones de la Guía para la Atención del Trabajo de Parto se consideraron completas. Los participantes estuvieron de acuerdo o muy de acuerdo con el diseño general, la estructura de la Guía para la Atención del Trabajo de Parto, y la utilidad de los umbrales de referencia para desencadenar evaluaciones y acciones adicionales. También estuvieron de acuerdo en que la Guía para la Atención del Trabajo de Parto tendrá un impacto positivo en la toma de decisiones clínicas y en el cuidado respetuoso. Los participantes no estuvieron de acuerdo con algunos parámetros propuestos en la Guía para la Atención del Trabajo de Parto, incluyendo el manejo de la situación por parte de las mujeres, parámetros urinarios y el estado neonatal.

**Conclusiones:**

Los futuros usuarios de la Guía para la Atención del Trabajo de Parto consideraron que las variables eran claras, pertinentes y apropiadas, y que, con pequeñas mejoras, podría tener un impacto positivo en la toma de decisiones clínicas y la atención respetuosa del parto.

## Résumé

**Contexte:**

Le partogramme est l'outil de suivi du travail le plus couramment utillis dans le monde. Cependant, il est utilisé de façon incorrecte ou non uniforme dans de nombreux contextes. En 2018, un groupe d'experts de l'OMS a examiné et révisé la conception du partogramme à la lumière de nouvelles données et a développé la première version du Guide de Gestion du Travail. L'objectif de cette étude était d'explorer les points de vue et les opinions du personnel de santé qualifié sur la première version du Guide de Gestion du Travail de l'OMS.

**Méthodes:**

Un vaste réseau mondial de recherche a permis d'identifier le personnel de santé qualifié (y compris des obstétriciens, des sages-femmes et des médecins généralistes) des deux sexes, en Afrique, Asie, Europe et en Amérique latine. Les coordinateurs nationaux du réseau ont invité entre 5 et 10 personnels de santé qualifiés de niveau intermédiaire à supérieur, et ayant travaillé dans des services de maternité à au cours des 5 dernières années. Un questionnaire en ligne, structuré, anonyme et à remplir soi-même, comprenant des questions fermées et ouvertes, a été conçu pour évaluer la clarté, la pertinence, la convenance de la fréquence d'enregistrement et l’intégrité des variables du Guide de Gestion du Travail.

**Résultats:**

Au total, 110 participants de 23 pays ont répondu à l'enquête entre décembre 2018 et janvier 2019. Les variables incluses dans le Guide de Gestion du Travail ont t généralement considérées claires, pertinentes et avec une fréquence d'enregistrement appropriée. La plupart des sections du Guide de Gestion du Travail sont considérées complètes. Les participants ont approuvé ou fortement approuvé le concept générale, la structure du Guide de Gestion du Travail et l'utilité des seuils de référence pour déclencher une évaluation et des actions supplémentaires. Ils ont également convenu que le Guide de Gestion du Travail aura potentiellement un impact positif sur les prise de décisions cliniques et les soins maternels respectueux. Les personnes interrogées ont exprimé leur désaccord avec la valeur de certaines variables, notamment la capacité d'adaptation, l'urine et l'état néonatal.

**Conclusions:**

Les futurs utilisateurs du le Guide de Gestion du Travail ont estimé que les variables étaient claires, pertinentes et appropriées, et qu'elles pourraient avoir un impact positif sur la prise de décision clinique et les soins de maternit respectueux, moyennant quelques améliorations mineures.

## Introduction

During the past 20 years, skilled birth attendance has been promoted widely to reduce preventable maternal and perinatal mortality and morbidity [1]. This has translated into large increases in both the coverage of births attended by skilled health personnel (62% in 2000 to 80% in 2017), and facility-based births [1]. However, these increases in coverage have not always translated into the expected reduction of maternal and perinatal mortality and morbidity during labour and childbirth, suggesting that suboptimal quality of care is still prevalent in health facilities [2–6]. Regular monitoring of labour and childbirth is vital to identifying risks or complications and to preventing adverse birth outcomes.

The partograph is the most commonly used labour monitoring tool, and it has been used for over 40 years by skilled health personnel providing care during labour. However, in light of the publication of the 2018 World Health Organization (WHO) recommendations on intrapartum care for a positive childbirth experience, the partograph required a revision to facilitate care according to emerging evidence and global priorities [7]. These WHO recommendations include new definitions and durations of the first and second stages of labour, and they highlight the importance of woman-centred care to optimize the experience of labour and childbirth for women and their babies.

WHO therefore initiated the development of a “next-generation” partograph, known as the WHO Labour Care Guide (LCG) (Additional file 1- First version of the LCG) with the following purposes: (a) continuously remind practitioners to offer supportive care throughout labour and childbirth, and remind them of what observations should be regularly made during labour to identify any emerging complication in mother and/or baby; (b) provide reference thresholds for abnormal labour observations that should trigger specific actions; (c) minimize over-diagnosis and under-diagnosis of abnormal labour events and the unnecessary use of interventions; and (d) support audits and quality of labour care improvement.

It is critical that the LCG includes appropriate parameters for labour monitoring and that it can meet the needs of maternity care providers. The findings from this research are intended to support a revision process that will improve the LCG. The objective of this study was to explore perspectives of skilled health personnel on the first version of the LCG.

## Methods

We conducted an international cross-sectional survey among skilled health personnel who were actively providing labour and childbirth care in a health facility. Participating skilled health personnel were asked about their opinions on the clarity, relevance, appropriateness of the frequency of recording, and the completeness of each section of the first version of the LCG. All participants consented to participating in the survey.

The first version of the LCG was organized into eight sections. The first section was for *identification*, specifically related to the time of diagnosis of active phase of labour, while the other sections were related to different categories of care provided throughout labour and childbirth: *supportive care* (companion, coping, pain relief, oral fluid and posture), *care of the baby* (baseline fetal heart rate (FHR), FHR deceleration, amniotic fluid, fetal position, caput and moulding), *care of the mother* (pulse, blood pressure, temperature and urine), *labour progress* (uterine contractions in 10 min, duration of contractions, cervix, and descent), *medication* (oxytocin, medicine, IV fluid), *shared decision-making* (assessment and plan), and *birth outcomes* (mode of birth, Apgar score at 5 min, blood loss, neonatal status and birthweight).

### Participants and sample

Skilled health personnel providing care during labour and childbirth (obstetricians, midwives and general practitioners) of any gender from four world regions—Africa, Asia, Europe and Latin America—were invited to participate. We targeted mid-level and senior providers who were currently practicing or had been working in labour wards during the previous 5 years. Eligible individuals were those who expressed their interest in participating, were fluent in English, French or Spanish, and provided consent to participate.

Participants were identified through the WHO Global Maternal Sepsis Study (GLOSS) research network [8]. Study coordinators from 23 countries of GLOSS research network, out of 30 invited, were involved in the selection and invitation of 5 to 10 skilled health personnel in their country/area of study. Country coordinators were encouraged to provide a diverse sample of skilled health personnel, based on the relevant cadres in their area.

### Procedures

A semi-structured questionnaire was designed, including closed and open-ended questions. The questionnaire was pretested among four maternal health researchers, using cognitive interviews for assessing its face-validity. Additionally, 12 English-proficient practicing obstetricians participated in a pilot study to assess length, clarity and relevance of the items of the questionnaire. Once the pilot was completed, a final revision of the instrument was made.

The online survey (via Survey Monkey™) was self-administered and anonymous (see Additional file 2: Online questionnaire). It assessed the clarity, relevance, and appropriateness of the frequency of recording and completeness of each of the eight sections of the LCG. The clarity and relevance of variables included in the LCG were rated on a 9-point Likert scale, ranging from 1 (very unclear or not relevant) to 9 (very clear and extremely relevant). The completeness of each section was assessed by asking participants if any other relevant variables should be added. Agreement regarding appropriateness of the frequency of recording was measured on a 4-point Likert scale: strongly agree, agree, disagree and strongly disagree. Participants were also asked to provide their views on the format of open-text variables in the LCG, and to provide comments on each section. Finally, there was a section for the general assessment of the LCG, including: providers’ opinions on the overall structure and organization of the LCG, clarity of instructions and abbreviations. The survey also asked about participants’ perceptions of its possible impact on clinical decision-making and respectful maternity care, and the usefulness of reference thresholds to trigger further assessment and necessary actions. The questionnaire was developed in English and translated into French and Spanish.

An invitation to participate in the survey was sent in December 2018 to 142 providers identified through GLOSS country coordinators. Among invited providers, 114 confirmed their interest and availability to participate in the online survey. Three reminders were sent to participants with partial or no responses over a 6-week period. Completeness and consistency between the survey items were monitored during survey administration.

### Data analysis

The median value and dispersion measures were computed for numerical variables, and proportions were calculated for categorical variables. For the items rated on a 9-point Likert scale, we measured the absolute and relative frequency in each of the three following intervals: low (1–3), intermediate (4–6) and high (7–9). Each item was classified as *inappropriate, uncertain* or *appropriate* based on the median rating and degree of disagreement (median 1 to 3 without disagreement = inappropriate; median 4 to 6 or any median with disagreement = uncertain; median 7 to 9 without disagreement = appropriate). Disagreement was considered present when RAND Disagreement Index (DI) ≥ 1.0. The DI is a continuous scale used to describe dispersion of participants´ ratings. A DI of 0 represents complete agreement while a DI ≥ 1 has been determined by RAND to indicate disagreement (see more details in Additional file 3: Methodological Details) [9]. Combined proportions were calculated and reported for the responses “Strongly agree” and “Agree”, “Strongly disagree” and “Disagree”.

Optional open-ended questions were located at the end of each section. Answers were analysed using a thematic analysis. Matrices were developed to facilitate comparison among responses and to organize the data by analytical themes. Main themes were the following: clarity, relevance and completeness. We also included comments that emerged from responses, regarding the tool design and layout. Finally, data was extracted and interpreted. All themes mentioned at least once were included.

## Results

Out of 114 skilled health personnel who confirmed their interest in participating and their availability, 110 across 23 countries completed the survey. Table 1 reports the characteristics of participants. Skilled health personnel represented professionals of a range of ages, professional background, and world regions: Africa (n = 53), Asia (n = 22), Latin America (n = 21) and Europe (n = 14). Female participants doubled the number of male participants. The majority had worked in labour wards and had used a partograph in the previous 5 years. There was a relatively larger number of African health care providers in the sample, as well as a larger number of obstetricians among all labour and birth attendants.

**Table 1:** Participants’ characteristics

Variable	N	%
	110	100
Region		
Africa	53	48
Asia	22	20
Latin America	21	19
Europe	14	13
Gender		
Female	75	71
Male	30	29
Age		
< 30	12	11
30–44	50	48
45–60	38	37
> 60	4	4
Profession		
Obstetrician	62	59
Midwife/nurse-midwife	34	32
OBGYN resident	4	4
General practitioner	3	3
Other	2	2
Time since qualification (years)		
< 5	17	17
5–20	61	59
> 20	25	24
Last time worked in labour ward (years)		
< 5	83	80
5–20	16	15
> 20	5	5
Last time used a partograph (years)		
< 5	89	86
5–20	13	13
> 20	1	1

Table 2 shows the median rating, the appropriateness classification, and the appropriateness of the frequency of recording of each variable. This table also describes median ratings and appropriateness classifications on reference thresholds proposed by the LCG for variables related to clinical parameters. Open-ended questions for each LCG section were optional and were only responded to by a proportion of the sample (between 16 and 40% depending on the section). Findings from open-ended questions are summarized in Additional file 4: Findings from open-ended questions.

**Table 2:** Assessment of LCG components

Variables (reference threshold)	Ratings on variables						Ratings on reference thresholds	
	Relevance		Clarity			Frequency of recording	Clarity	
	Median rating	Appropriateness classification	Median rating	Appropriateness classification		Strongly agree or agree n (%)	Median rating	Appropriateness classification
Section 1: Identification								
Parity	9	Appropriate	9	Appropriate		N/A	N/A	N/A
Labour onset	9	Appropriate	9	Appropriate		N/A	N/A	N/A
Active labour diagnosis	9	Appropriate	9	Appropriate		N/A	N/A	N/A
Ruptured membranes	9	Appropriate	9	Appropriate		N/A	N/A	N/A
Risk factor	9	Appropriate	9	Appropriate		N/A	N/A	N/A
Section 2: Supportive care								
Companion	9	Appropriate	9		Appropriate	90 (84)	N/A	N/A
Coping	8	Uncertain	7		Uncertain	75 (70)	N/A	N/A
Pain relief	9	Appropriate	9		Appropriate	95 (89)	N/A	N/A
Oral fluid	9	Appropriate	9		Appropriate	89 (83)	N/A	N/A
Posture	8	Appropriate	8		Appropriate	80 (75)	N/A	N/A
Section 3: Care of the baby								
Baseline FHR (< 110, ≥ 160)	9	Appropriate	9		Appropriate	96 (90)	9	Appropriate
FHR deceleration (L)	9	Appropriate	9		Appropriate	99 (93)	9	Appropriate
Amniotic fluid (M+++)	9	Appropriate	9		Appropriate	96 (90)	9	Appropriate
Fetal position (OP, O)	9	Appropriate	9		Appropriate	83 (78)	9	Appropriate
Caput (+++)	9	Appropriate	9		Appropriate	80 (75)	9	Appropriate
Moulding (+++)	9	Appropriate	9		Appropriate	73 (69)	9	Appropriate
Section 4: Care of the mother								
Pulse (< 60, ≥ 120)	9	Appropriate	9		Appropriate	91 (86)	9	Appropriate
Systolic BP (< 80, ≥ 140)	9	Appropriate	9		Appropriate	86 (81)	9	Appropriate
Diastolic BP (≥ 90)	9	Appropriate	9		Appropriate	87 (82)	9	Appropriate
Temperature ºC (< 35, ≥ 37.5)	9	Appropriate	9		Appropriate	76 (72)	9	Appropriate
Urine (P++, A++)	9	Appropriate	9		Appropriate	63 (59)	9	Appropriate
Section 5: Labour progress								
Contractions per 10 min (≤ 2, > 5)	9	Appropriate	9		Appropriate	97 (92)	9	Appropriate
Duration of contractions (< 20, > 60)	9	Appropriate	9		Appropriate	95 (90)	9	Appropriate
Cervix recorded as 5–10 cm (≥ 2 h to ≥ 6 h)	9	Appropriate	9		Appropriate	81 (82)	9	Uncertain
Descendent	9	Appropriate	9		Appropriate	89 (85)		
Section 6: Medication								
Oxytocin	9	Appropriate	9		Appropriate	93 (89)	N/A	N/A
Medicine	9	Appropriate	9		Appropriate	92 (88)	N/A	N/A
IV fluid	9	Appropriate	9		Appropriate	92 (88)	N/A	N/A
Section 7: Shared decision-making								
Assessment	9	Appropriate	9		Appropriate	90 (86)	N/A	N/A
Plan	9	Appropriate	9		Appropriate	91 (87)	N/A	N/A
Section 8: Birth outcomes								
Mode of birth	9	Appropriate	9		Appropriate	N/A	N/A	N/A
Apgar score at 5 min	9	Appropriate	9		Appropriate	N/A	N/A	N/A
Blood loss	9	Appropriate	9		Appropriate	N/A	N/A	N/A
Neonatal status	9	Appropriate	9		Appropriate	N/A	N/A	N/A
Birthweight	9	Appropriate	9		Appropriate	N/A	N/A	N/A

For Section 1 (*Identification*), all variables received a median score of 9 with appropriate classification of rating for clarity and relevance. Overall, participants perceived all the variables in this section to be clear and relevant, and there was agreement that the open text format for recording “Parity”, “Labour onset” and “Risk factor” was appropriate. However, 55% of the participants agreed that one or more variables (e.g. date and time of admission) should be added to this section to make it complete. Regarding open-ended questions, participants required clearer definitions of “labour onset” (also categories—such as “induced” or “spontaneous”) and “risk factors”. The potential difficulties in registering the start of active phase if the women was admitted late in labour were also reported. It was suggested to include maternal and fetal clinical variables and administrative information for women´ follow-up.

For Section 2 (*Supportive care*), the majority of variables had high median ratings for clarity and relevance (i.e. 8–9) and were considered appropriate, with the exception of the variable “Coping”. “Coping” had a median value of 7 without agreement for clarity. Assessment of relevance showed that participants considered each variable appropriate, but consensus on the relevance of including the whole section (Supportive Care) in the LCG and the variable “coping” was not reached. Provider perspectives on including the “Supportive care” section were largely similar when stratified by cadre (36% and 39% of physicians and midwives respectively would not include this whole section). Participants agreed with the frequency of recording this section’s variables, except for “coping” and “posture”, where 30% and 25% of participants disagreed with the proposed frequency of recording, respectively. Most of participants were in favour of less frequent recording. From the open-ended question, participants reported lack of clarity or problematic terms, such as “companionship”. Lack of clarity on how to record pain relief was described (type of analgesia, epidural, pharmacological or not pharmacological), and some participants preferred to record "effective pain relief ". For the variable “posture”, participants suggested new abbreviations for categories, “walking” for example, and highlighted that “SP” (for supine position) should be included in the abbreviations section.

Participants agreed with the clarity, relevance, completeness, and appropriateness of Section 3 (*Care of the baby*), as all variables received high median values with agreement. With respect to the appropriateness of the frequency of recording variables, a high proportion of agreement was observed for “baseline fetal heart rate” (FHR), “FHR decelerations” and “amniotic fluid”. A lower level of agreement was observed for “fetal position”, “caput” and “moulding”, with the majority of participants favouring a lower frequency of recording. Participants also agreed that reference thresholds were clear. In the open-ended question, participants made suggestions to improve the recording of some variables such as FHR deceleration, caput succedaneum and moulding, and the frequency for recording.

Section 4 (*Care of the mother*) obtained very high ratings for relevance, clarity and clarity of reference threshold, and were considered appropriate. A lower proportion of agreement was found for the frequency of recording of “urine” as 41% of the participants were in favour of recording it less frequently. In open-ended questions, participants made suggestions for variables such as pulse, blood pressure and urine.

Sections 5 (*Labour progress*), 6 (*Medication*), 7 (*Shared decision-making*) and 8 (*Birth outcomes*) obtained high ratings on all assessed criteria. “Cervix” from Section 5 was the only variable of the section that obtained a lower proportion of high ratings on clarity (71%) and on the clarity of its reference threshold (67%)—this last assessment showed dissent among participants. The Section 7 variable “assessment” also received a slightly lower proportion of high ratings for clarity. The section 8 variable “neonatal status” obtained a lower level of agreement on the proposed format for recording.

In the Section 6 open-ended questions, participants suggested a better explanation of how to record medications, type of intravenous (IV) fluid being reported, and adding abbreviations to register the use of oxytocin. Some participants suggested including a variable to record “use of oxygen”. In Section 7 there were some difficulties in understanding the difference between “Assessment” and “Plan”. While in Section 8, some providers suggested to include variables such as Apgar at 1, 5 and 10 min, newborn sex, any abnormality, and interventions at third stage of labour.

Regarding additional variables required per section, the completeness was lower in Sections 1 and 8, where 55% and 42% of participants respectively, considered that additional variables needed to be added (results shown in Table 3).

**Table 3:** Completeness of LCG sections

Sections	Additional variables are required	
	N	%
	110	100
Section 1: Identification	60	55
Section 2: Supportive care	21	20
Section 3: Care of the baby	21	20
Section 4: Care of the mother	10	10
Section 5: Labour progress	14	13
Section 6: Medication	11	11
Section 7: Shared decision-making	8	8
Section 8: Birth outcomes	44	42

Finally, the overall assessment of the LCG received high levels of agreement regarding its potential to lead to a positive impact on quality of care: would facilitate decision-making (96%), and implementation of respectful maternity care (94%), sections organization (93%), and general design—clear instructions (80%), clear abbreviations (84%). However, 28% of participants reported that it would not be easy to complete (Table 4).

**Table 4:** General assessment of the LCG

To what extent do you agree with the following statements about the LCG?	Agree or strongly agree	
	N	%
	110	100
It will facilitate clinical decision-making	100	96
The reference thresholds will trigger further assessment and necessary action	98	93
It will facilitate implementation of respectful maternity care policy	99	94
Contains all relevant variables	90	85
It will be easy to complete	76	72
Instructions are clear	84	80
Abbreviations are clear	89	84
The sections are logically organized	98	93
24-h time format is very appropriate	94	89
Enables provider identification	85	81

## Discussion

This publication describes the findings of an international online survey of skilled health personnel in 23 countries on the first version of the LCG, a new partograph that was under development by WHO. Understanding the views of these practitioners was meant to inform the revisions and improvements of the LCG.

In general, variables included in the LCG were considered clear, relevant and to have an appropriate frequency of recording. In addition, most sections were considered complete for labour monitoring. The LCG received high levels of agreement regarding its potential for making a positive impact on quality of care, the organization of sections, organization and general design. Few variables received low ratings, or agreements among participants were not achieved.

This study provided useful information regarding how to improve the LCG design, and for the development of an LCG user’s manual and training materials. Regarding reference threshold for cervical dilatation, in recent years, it was demonstrated that the progress of 1 cm/h is unrealistic for many women in labour, and that the length of the dilatation period does not correlate with adverse perinatal events when observations of other maternal and perinatal health parameters remain normal [10, 11]. In light of new evidence, previous alert and action lines used in labour monitoring for several decades are no longer recommended [12]. The response of the participants suggest that some of them were not familiar or aware of the new WHO recommendations and underpinning evidence on this subject.

Health care professionals showed uncertainty (no agreement) on including Section 2 (*Supportive care*) in the LCG. This could be related to the fact that the previous partograph designs historically had been used to monitor only clinical variables that are highly valued by health care personnel providing care during labour and childbirth. A new feature of the LCG is the inclusion of non-clinical observations (such as the ones included in the “*Supportive Care*" section) as part of labour monitoring. This update is supported by current WHO recommendations and the corresponding WHO model for intrapartum care, which promotes woman-centred care [7]. The uncertainty (no agreement) on the clarity of one specific item of the LCG is noteworthy: “coping”. “Coping” was considered unclear in all three languages; although it was less clear for Spanish and French-speakers in comparison to English-speakers. Overall, there are challenges to measure this construct given that classic 0–10 pain assessment scales may be inadequate during labour [9].

Two sections, 1 (*Identification and labour admission characteristics*) and 8 (*Birth outcomes*), were considered less complete compared to the rest. Unlike Sections 2–7, Sections 1 and 8 do not include variables that monitor the use of the recommendations for labour and childbirth care. Although a large proportion of participants believed it was necessary to add more variables to these sections, they are variables that are traditionally recorded in medical records. The LCG is not intended to replace medical records but rather it is meant to be a tool to help providers implement the WHO intrapartum care recommendations.

The LCG provides guidance on the frequency of assessing observations for early detection of complications and to avoid unnecessary interventions (such as repeated vaginal examinations). Some health care professionals did not agree on the frequency of assessments proposed. We learned from this study that further clarifications should be provided throughout supporting materials to appropriately guide clinicians on the frequency of assessments. Although the LCG suggests some guidance on the frequency of assessments, this will mainly depend on a comprehensive assessment of the well-being of each woman and baby, and on the local context (e.g. level of care).

This study has several strengths: (a) the survey was pretested (to ensure face validity) and piloted; (b) the participants were international and included a diverse sample of experienced skilled health personnel; and (c) the questionnaire included both close and open-ended questions, enriching the results with qualitative data. Nonetheless, this study had some limitations. Although the survey was administrated in three languages (English, French and Spanish), selection bias could possibly have been introduced given that certain professional cadres (e.g. nurses, midwives) might only speak their native language.

Participating skilled health personnel considered the components of the LCG relevant, clear and appropriate. However, the LCG design alone cannot address systemic issues. The 2017 realist review by Bedwell et al. showed that, in order to use a partograph effectively, providers need additional supports: essential equipment, clear hospital policies on correct partograph use, effective supervision, and regular refresher training [13]. Although our findings will be used to revise and improve the LCG, research on the implementation of the LCG is needed to have a positive impact on effective labour care.

## Conclusion

Skilled health personnel from several countries and regions largely considered the components of the WHO Labour Care Guide relevant, clear and appropriate. While their opinions were supportive of this new labour monitoring tool, they identified a few areas where the LCG could be improved or clarified in order to facilitate its adoption and use. The development of supporting and training materials will be required to guide health professionals on how to use the LCG. Further evaluation to assess the usability, feasibility, acceptability of LCG in clinical settings is warranted.

## Supplementary Information

**Additional file 1.** First version of the LCG.

**Additional file 2.** Online questionnaire.

**Additional file 3.** Methodological details.

**Additional file 4.** Findings from open-ended questions.

## Data Availability

Full de-identified datasets are available upon request from the corresponding author.

## Abbreviations

CONICET: Consejo Nacional de Investigaciones Científicas y Técnicas
DI: Disagreement Index
FHR: Fetal heart rate
GLOSS: WHO Global Maternal Sepsis Study
HRP: UNDP/UNFPA/UNICEF/WHO/World Bank Special Programme of Research, Development and Research Training in Human Reproduction
IECS: Institute for Clinical Effectiveness and Health Policy
IV: Intravenous
LCG: Labour Care Guide
SP: Supine position
UNDP: United Nations Development Programme
UNFPA: United Nations Population Fund
UNICEF: United Nations Children’s Fund
WHO: World Health Organization
