# Supplementary material for: The development of the WHO Labour Care Guide: an international survey of maternity care providers
Source: Reprod Health. 2021 Mar 22;18:66. doi: 10.1186/s12978-021-01074-2 (PMC7986022; doi:10.1186/s12978-021-01074-2)
Supplement: Supplementary file 1 — Additional file 1. First version of the LCG. [file 12978_2021_1074_MOESM1_ESM.docx]

# Additional file 1: first version of the WHO Labour Care Guide (LC
