# Supplementary material for: The development of the WHO Labour Care Guide: an international survey of maternity care providers
Source: Reprod Health. 2021 Mar 22;18:66. doi: 10.1186/s12978-021-01074-2 (PMC7986022; doi:10.1186/s12978-021-01074-2)
Supplement: Supplementary file 2 — Additional file 2. Online questionnaire. [file 12978_2021_1074_MOESM2_ESM.docx]

# Additional file 2 : online questionnaire


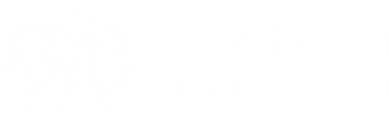


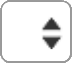

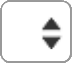

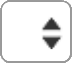

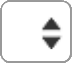

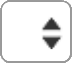

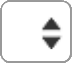

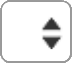

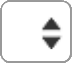

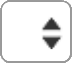

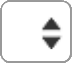

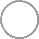

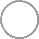

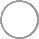

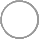


| INTERNATIONAL CONSULTATION ON THE NEW WHO PARTOGRAPH |
| --- |
| **Section 1: Woman identification and labour admission characteristics** |
| About this section: This section aims to capture the most important maternal demographic and obstetric information that is essential for labour monitoring and action. Other details are expected to be included in the woman’s medical record. [Click here to see Section 1](https://www.dropbox.com/s/9oafzzy7wbw1hzy/PDF_Section%201_eng.pdf?dl=0) this section highlighted on the new partograph.  1. **Survey questions: Please rate each of the variables below with the option that best represents your opinion.**   Please rate your opinion using a 9-point scale.  Is each term easy to understand as presented How relevant is each variables for labour in this section? Consider 1=very unclear; management? Consider 1=not relevant;  9=very clear 9=extremely relevant Parity  Labour onset  Active labour diagnosis (Date; Time) Ruptured membranes (Date; Time) Risk factor  To what extent do you agree with recording **Parity** in an open text? [Click heretoseesection1](https://www.dropbox.com/s/9oafzzy7wbw1hzy/PDF_Section%201_eng.pdf?dl=0) .  Strongly disagree Disagree Agree Strongly agree |


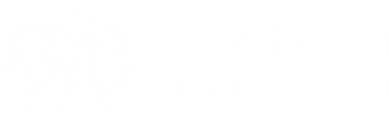


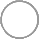

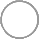

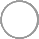

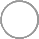


| INTERNATIONAL CONSULTATION ON THE NEW WHO PARTOGRAPH | |
| --- | --- |
| **Section 1: Woman identification and labour admission characteristics** | |
| Would you rather register data using a checkbox for **nulliparous/multiparous**? [Click heretoseesectio](https://www.dropbox.com/s/9oafzzy7wbw1hzy/PDF_Section%201_eng.pdf?dl=0)  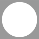 Yes  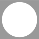 No (please specify)  To what extent do you agree with recording **Labour Onset** in an open text? [Click heretoseesection1](https://www.dropbox.com/s/9oafzzy7wbw1hzy/PDF_Section%201_eng.pdf?dl=0) .  Strongly disagree Disagree Agree | [n1](https://www.dropbox.com/s/9oafzzy7wbw1hzy/PDF_Section%201_eng.pdf?dl=0) .  Strongly agree |


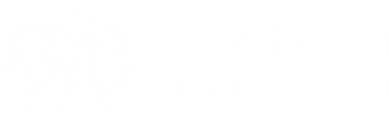


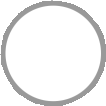

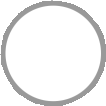

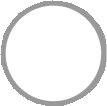

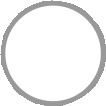


| INTERNATIONAL CONSULTATION ON THE NEW WHO PARTOGRAPH | |
| --- | --- |
| **Section 1: Woman identification and labour admission characteristics** | |
| Would you rather register data using a checkbox for **spontaneous/induced**?[Click heretoseesection1](https://www.dropbox.com/s/9oafzzy7wbw1hzy/PDF_Section%201_eng.pdf?dl=0)  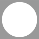 Yes  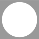 No (please specify)  To what extent do you agree with recording **Risk Factors** in an open text? [Click heretoseesection1](https://www.dropbox.com/s/9oafzzy7wbw1hzy/PDF_Section%201_eng.pdf?dl=0) .  Strongly disagree Disagree Agree | .  Strongly agree |


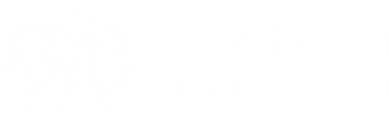


| INTERNATIONAL CONSULTATION ON THE NEW WHO PARTOGRAPH |
| --- |
| **Section 1: Woman identification and labour admission characteristics** |
| How would you rather register Risk factor? [Click heretoseesection1](https://www.dropbox.com/s/9oafzzy7wbw1hzy/PDF_Section%201_eng.pdf?dl=0) . 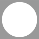 Numerical codes  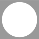 Yes/No, and an open text 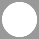 Other (please specify)  Is there any additional demographic or obstetric variable that you would consider as very relevant (rated 7 to 9 on a 9-point scale) for addition in this section? [Click heretoseesection1](https://www.dropbox.com/s/9oafzzy7wbw1hzy/PDF_Section%201_eng.pdf?dl=0) .  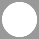 Yes 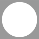 No |


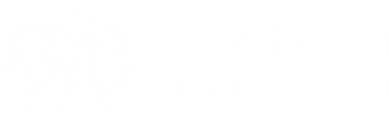


| INTERNATIONAL CONSULTATION ON THE NEW WHO PARTOGRAPH |
| --- |
| **Section 1: Woman identification and labour admission characteristics** |
| Which if any of the following variables would you consider as very relevant for inclusion in this section? You can select one or more options. [Click here to see section 1](https://www.dropbox.com/s/9oafzzy7wbw1hzy/PDF_Section%201_eng.pdf?dl=0) .  Gravida Hospital name  Date of admission Time of admission Other (please specify)  Please use the box below to provide any additional comments or suggestions you may have on this section. |


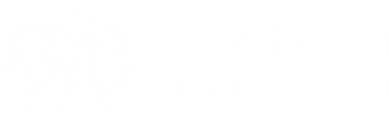


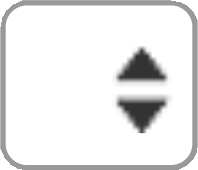

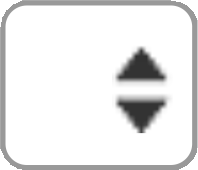

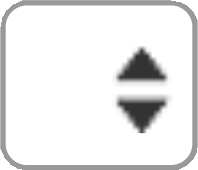

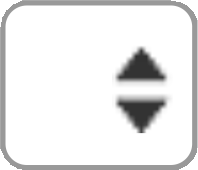

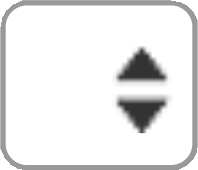

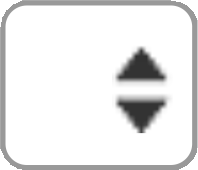

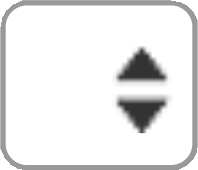

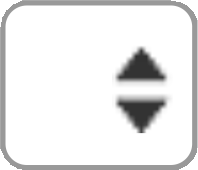

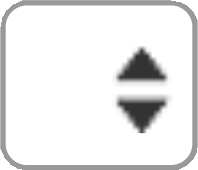

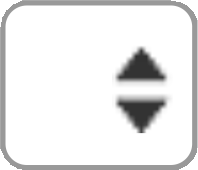

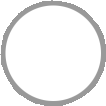

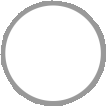

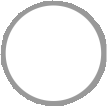

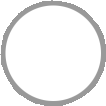

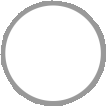

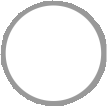

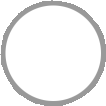

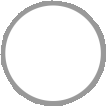

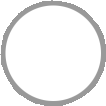

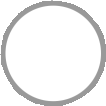

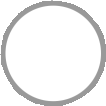

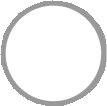

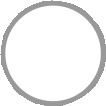


| INTERNATIONAL CONSULTATION ON THE NEW WHO PARTOGRAPH |
| --- |
| **Section 2: Supportive care** |
| 1. **About this section: This section aims to encourage the consistent practice of supportive, woman-centered, and respectful care throughout labour and childbirth.** [**Click here to see section 2**](https://www.dropbox.com/s/ks1m7q6s3lt26fj/PDF_Section%202_eng.pdf?dl=0) **on the new partograph.** 2. **Survey questions: Please rate each of the variables below with the option that best represents your opinion.**   Please rate your opinion using a 9-point scale.  Is each term easy to understand as presented How relevant is each variable for labour in this section? Consider 1=very unclear; management? Consider 1=not relevant;  9=very clear 9=extremely relevant Companion  Coping Pain relief Oral fluid Posture  How relevant would you rate having this whole section being included in the partograph? Please rate your opinion using a 9-point scale, where 1 represents not relevant and 9 extremely relevant.  1 2 3 4 5 6 7 8 9  To what extent do you agree with the frequency **Companion** is being recorded?  Strongly disagree Disagree Agree Strongly agree |


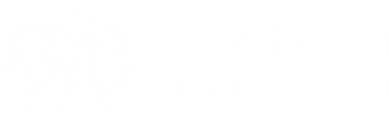


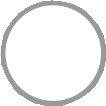

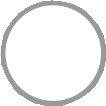

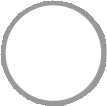

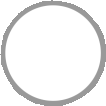


| INTERNATIONAL CONSULTATION ON THE NEW WHO PARTOGRAPH | |
| --- | --- |
| **Section 2: Supportive care** | |
| How often would you record **Companion**? [Click heretoseesection2](https://www.dropbox.com/s/ks1m7q6s3lt26fj/PDF_Section%202_eng.pdf?dl=0) . 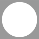 More frequently  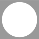 Less frequently  To what extent do you agree with the frequency **Coping** is being recorded? [Click heretoseesect](https://www.dropbox.com/s/ks1m7q6s3lt26fj/PDF_Section%202_eng.pdf?dl=0)  Strongly disagree Disagree Agree | [ion2](https://www.dropbox.com/s/ks1m7q6s3lt26fj/PDF_Section%202_eng.pdf?dl=0) .  Strongly agree |


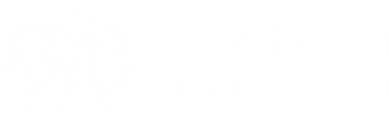


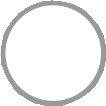

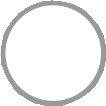

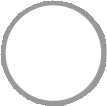

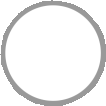


| INTERNATIONAL CONSULTATION ON THE NEW WHO PARTOGRAPH |
| --- |
| **Section 2: Supportive care** |
| How often would you record **Coping**? [Click heretoseesection2](https://www.dropbox.com/s/ks1m7q6s3lt26fj/PDF_Section%202_eng.pdf?dl=0) . 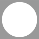 More frequently  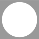 Less frequently  To what extent do you agree with the frequency **Pain Relief** is being recorded? [Click heretoseesection2](https://www.dropbox.com/s/ks1m7q6s3lt26fj/PDF_Section%202_eng.pdf?dl=0) .  Strongly disagree Disagree Agree Strongly agree |


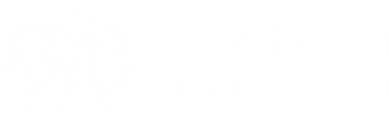


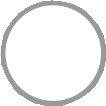

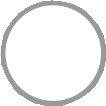

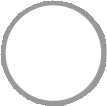

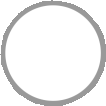


| INTERNATIONAL CONSULTATION ON THE NEW WHO PARTOGRAPH | |
| --- | --- |
| **Section 2: Supportive care** | |
| How often would you record **Pain Relief**? [Click heretoseesection2](https://www.dropbox.com/s/ks1m7q6s3lt26fj/PDF_Section%202_eng.pdf?dl=0) . 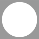 More frequently  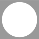 Less frequently  To what extent do you agree with the frequency **Oral Fluid** is being recorded? [Click heretoseese](https://www.dropbox.com/s/ks1m7q6s3lt26fj/PDF_Section%202_eng.pdf?dl=0)  Strongly disagree Disagree Agree | [ction2](https://www.dropbox.com/s/ks1m7q6s3lt26fj/PDF_Section%202_eng.pdf?dl=0) .  Strongly agree |


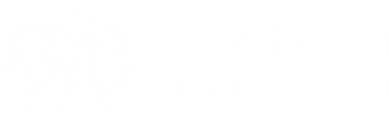


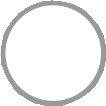

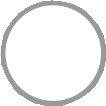

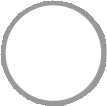

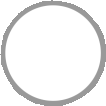


| INTERNATIONAL CONSULTATION ON THE NEW WHO PARTOGRAPH | |
| --- | --- |
| **Section 2: Supportive care** | |
| How often would you record **Oral Fluid**? [Click heretoseesection2](https://www.dropbox.com/s/ks1m7q6s3lt26fj/PDF_Section%202_eng.pdf?dl=0) . 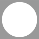 More frequently  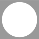 Less frequently  To what extent do you agree with the frequency **Posture** is being recorded? [Click heretoseesec](https://www.dropbox.com/s/ks1m7q6s3lt26fj/PDF_Section%202_eng.pdf?dl=0)  Strongly disagree Disagree Agree | [tion2](https://www.dropbox.com/s/ks1m7q6s3lt26fj/PDF_Section%202_eng.pdf?dl=0) .  Strongly agree |

| INTERNATIONAL CONSULTATION ON THE NEW WHO PARTOGRAPH |
| --- |
| **Section 2: Supportive care** |
| How often would you record **Posture**? [Click heretoseesection2](https://www.dropbox.com/s/ks1m7q6s3lt26fj/PDF_Section%202_eng.pdf?dl=0) .  More frequently Less frequently  Is there any additional observation relating to supportive care that you would consider as very relevant (rated 7 to 9 on a 9-point scale for inclusion in this section?  No  Yes (please specify)  Please use the box below to provide any additional comments or suggestions you may have on this section. |


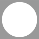


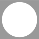


)


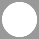


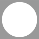


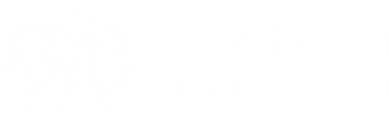


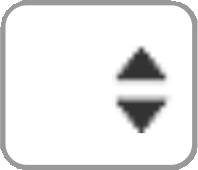

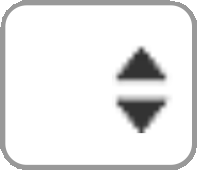

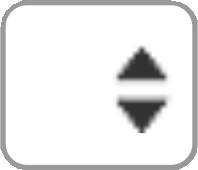

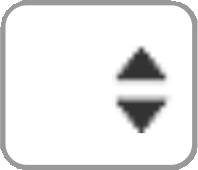

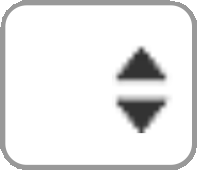

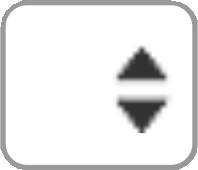

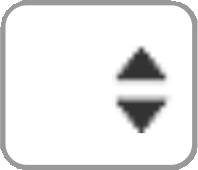


| INTERNATIONAL CONSULTATION ON THE NEW WHO PARTOGRAPH |
| --- |
| **Section 3: Care of the baby** |
| 1. **About this section: This section aims to encourage the consistent practice of intermittent monitoring of fetal wellbeing.**   [**Click here to see this section 3**](https://www.dropbox.com/s/v0sqwk8r73obltd/PDF_Section%203_eng.pdf?dl=0) **on the new partograph.**   1. **Survey questions: Please rate each of the variables below with the option that best represents your opinion.**   Please rate your opinion using a 9-point scale. Please note that the values of the alerts are those in parentheses; FHR=Fetal heart rate.  Is each term easy to Is each value easy to How relevant is each variable understand as presented in understand as presented in the for labour management?  this section? Consider 1=very alert column? 1=very unclear; Consider 1=not relevant; unclear; 9=very clear 9=very clear 9=extremely relevant  Baseline FHR (<110, ≥160) FHR deceleration (L) Amniotic fluid (M +++)  Fetal position (OP, O) Caput (+++)  Moulding (+++)  To what extent do you agree with the frequency **Baseline FHR** is being recorded? [Click heretoseesection3](https://www.dropbox.com/s/v0sqwk8r73obltd/PDF_Section%203_eng.pdf?dl=0) .  Strongly disagree Disagree Agree Strongly agree |

| INTERNATIONAL CONSULTATION ON THE NEW WHO PARTOGRAPH |
| --- |
| **Section 3: Care of the baby** |
| How often would you record **Baseline FHR**? [Click heretoseesection3](https://www.dropbox.com/s/v0sqwk8r73obltd/PDF_Section%203_eng.pdf?dl=0) . More frequently  Less frequently  To what extent do you agree with the frequency **FHR deceleration** is being recorded? [Click heretoseesection3](https://www.dropbox.com/s/v0sqwk8r73obltd/PDF_Section%203_eng.pdf?dl=0) .  Strongly disagree Disagree Agree Strongly agree |

| INTERNATIONAL CONSULTATION ON THE NEW WHO PARTOGRAPH |
| --- |
| **Section 3: Care of the baby** |
| How often would you record **FHR deceleration** ? [Click heretoseesection3](https://www.dropbox.com/s/v0sqwk8r73obltd/PDF_Section%203_eng.pdf?dl=0) . More frequently  Less frequently  To what extent do you agree with the frequency **Amniotic fluid** is being recorded? [Click heretoseesection3](https://www.dropbox.com/s/v0sqwk8r73obltd/PDF_Section%203_eng.pdf?dl=0) .  Strongly disagree Disagree Agree Strongly agree |

| INTERNATIONAL CONSULTATION ON THE NEW WHO PARTOGRAPH |
| --- |
| **Section 3: Care of the baby** |
| How often would you record **Amniotic fluid**? [Click heretoseesection3](https://www.dropbox.com/s/v0sqwk8r73obltd/PDF_Section%203_eng.pdf?dl=0) . More frequently  Less frequently  To what extent do you agree with the frequency **Fetal position** is being recorded? [Click heretoseesection3](https://www.dropbox.com/s/v0sqwk8r73obltd/PDF_Section%203_eng.pdf?dl=0) .  Strongly disagree Disagree Agree Strongly agree |

| INTERNATIONAL CONSULTATION ON THE NEW WHO PARTOGRAPH | |
| --- | --- |
| **Section 3: Care of the baby** | |
| How often would you record **Fetal position**? [Click heretoseesection3](https://www.dropbox.com/s/v0sqwk8r73obltd/PDF_Section%203_eng.pdf?dl=0) . More frequently  Less frequently  To what extent do you agree with the frequency **Caput** is being recorded? [Click heretoseesectio](https://www.dropbox.com/s/v0sqwk8r73obltd/PDF_Section%203_eng.pdf?dl=0)  Strongly disagree Disagree Agree | [n3](https://www.dropbox.com/s/v0sqwk8r73obltd/PDF_Section%203_eng.pdf?dl=0) .  Strongly agree |

| INTERNATIONAL CONSULTATION ON THE NEW WHO PARTOGRAPH | |
| --- | --- |
| **Section 3: Care of the baby** | |
| How often would you record **Caput**? [Click heretoseesection3](https://www.dropbox.com/s/v0sqwk8r73obltd/PDF_Section%203_eng.pdf?dl=0) . More frequently  Less frequently  To what extent do you agree with the frequency **Moulding** is being recorded? [Click heretoseese](https://www.dropbox.com/s/v0sqwk8r73obltd/PDF_Section%203_eng.pdf?dl=0)  Strongly disagree Disagree Agree | [ction3](https://www.dropbox.com/s/v0sqwk8r73obltd/PDF_Section%203_eng.pdf?dl=0) .  Strongly agree |

| INTERNATIONAL CONSULTATION ON THE NEW WHO PARTOGRAPH |
| --- |
| **Section 3: Care of the baby** |
| How often would you record **Moulding**? [Click heretoseesection3](https://www.dropbox.com/s/v0sqwk8r73obltd/PDF_Section%203_eng.pdf?dl=0) . More frequently  Less frequently  Is there any additional variable that you would consider as very relevant (rated 7 to 9 on a 9-point scale) for inclusion in this section? No  Yes (please specify)  Please use the box below to provide any additional comments or suggestions you may have on this section. |

| INTERNATIONAL CONSULTATION ON THE NEW WHO PARTOGRAPH |
| --- |
| **Section 4: Care of the mother** |
| 1. **About this section: This section aims to encourage the consistent practice of intermittent monitoring of maternal well- being.** [**Click here to see section 4**](https://www.dropbox.com/s/q3mr7tuazmazip0/PDF_Section%204_eng.pdf?dl=0) **on the new partograph** 2. **Survey questions: Please rate each of the variables below with the option that best represents your opinion.**   Please rate your opinion using a 9-point scale. Please note that the values of the alerts are those in parentheses.  Is each term easy to Is each value easy to How relevant is each variable understand as presented in understand as presented in the for labour management?  this section? Consider 1=very alert column? 1=very unclear; Consider 1=not relevant; unclear; 9=very clear 9=very clear 9=extremely relevant  Pulse (<60, ≥120) Systolic BP (<80, ≥140) Diastolic BP (≥90)  Temperature ºC (<35, ≥ 37.5) Urine (P++, A++)  To what extent do you agree with the frequency **Pulse** is being recorded?  Strongly disagree Disagree Agree Strongly agree |

| INTERNATIONAL CONSULTATION ON THE NEW WHO PARTOGRAPH | |
| --- | --- |
| **Section 4: Care of the mother** | |
| How often would you record **Pulse**? [Click heretoseesection4](https://www.dropbox.com/s/q3mr7tuazmazip0/PDF_Section%204_eng.pdf?dl=0) . More frequently  Less frequently  To what extent do you agree with the frequency **Systolic BP** is being recorded? [Click heretosee](https://www.dropbox.com/s/q3mr7tuazmazip0/PDF_Section%204_eng.pdf?dl=0)  Strongly disagree Disagree Agree | [section4](https://www.dropbox.com/s/q3mr7tuazmazip0/PDF_Section%204_eng.pdf?dl=0) .  Strongly agree |

| INTERNATIONAL CONSULTATION ON THE NEW WHO PARTOGRAPH |
| --- |
| **Section 4: Care of the mother** |
| How often would you record **Systolic BP**? [Click heretoseesection4](https://www.dropbox.com/s/q3mr7tuazmazip0/PDF_Section%204_eng.pdf?dl=0) . More frequently  Less frequently  To what extent do you agree with the frequency **Diastolic BP** is being recorded? [Click heretoseesection4](https://www.dropbox.com/s/q3mr7tuazmazip0/PDF_Section%204_eng.pdf?dl=0) .  Strongly disagree Disagree Agree Strongly agree |

| INTERNATIONAL CONSULTATION ON THE NEW WHO PARTOGRAPH |
| --- |
| **Section 4: Care of the mother** |
| How often would you record **Diastolic BP**? [Click heretoseesection4](https://www.dropbox.com/s/q3mr7tuazmazip0/PDF_Section%204_eng.pdf?dl=0) . More frequently  Less frequently  To what extent do you agree with the frequency **Temperature ºC** is being recorded? [Click heretoseesection4](https://www.dropbox.com/s/q3mr7tuazmazip0/PDF_Section%204_eng.pdf?dl=0) .  Strongly disagree Disagree Agree Strongly agree |

| INTERNATIONAL CONSULTATION ON THE NEW WHO PARTOGRAPH | |
| --- | --- |
| **Section 4: Care of the mother** | |
| How often would you record **Temperature ºC**? [Click heretoseesection4](https://www.dropbox.com/s/q3mr7tuazmazip0/PDF_Section%204_eng.pdf?dl=0) . More frequently  Less frequently  To what extent do you agree with the frequency **Urine** is being recorded? [Click heretoseesection](https://www.dropbox.com/s/q3mr7tuazmazip0/PDF_Section%204_eng.pdf?dl=0)  Strongly disagree Disagree Agree | [4](https://www.dropbox.com/s/q3mr7tuazmazip0/PDF_Section%204_eng.pdf?dl=0) .  Strongly agree |

| INTERNATIONAL CONSULTATION ON THE NEW WHO PARTOGRAPH |
| --- |
| **Section 4: Care of the mother** |
| How often would you record **Urine**? [Click heretoseesection4](https://www.dropbox.com/s/q3mr7tuazmazip0/PDF_Section%204_eng.pdf?dl=0) . More frequently  Less frequently  Is there any additional variable that you would consider as very relevant (rated 7 to 9 on a 9-point scale) for inclusion in this section? No  Yes (please specify)  Please use the box below to provide any additional comments or suggestions you may have on this section. |

| INTERNATIONAL CONSULTATION ON THE NEW WHO PARTOGRAPH |
| --- |
| **Section 5: Labour progress** |
| 1. **About this section: This section aims to encourage the consistent practice of intermittent monitoring of labour progression parameters.** [**Click here to see section 5**](https://www.dropbox.com/s/ppas54rlqswvam0/PDF_Section%205_eng.pdf?dl=0) **on the new partograph** 2. **Survey questions: Please rate each of the variables below with the option that best represents your opinion.**   Please rate your opinion using a 9-point scale. Please note that the values of the alerts are those in parentheses.  Is each term easy to Is each value easy to How relevant is each variable understand as presented in understand as presented in the for labour management?  this section? Consider 1=very alert column? 1=very unclear; Consider 1=not relevant; unclear; 9=very clear 9=very clear 9=extremely relevant  Contractions per 10 min (≤2, >5)  Duration of contractions (<20, >60) Cervix recorded as 5-10cm (≥ 2h to ≥  6h)  Descendent recorded as 0, 1, 2, 3, 4, 5  To what extent do you agree with the frequency **Contractions per 10 min** is being recorded?  Strongly disagree Disagree Agree Strongly agree |

| INTERNATIONAL CONSULTATION ON THE NEW WHO PARTOGRAPH |
| --- |
| **Section 5: Labour progress** |
| How often would you record **Contractions per 10 min**? [Click heretoseesection5](https://www.dropbox.com/s/ppas54rlqswvam0/PDF_Section%205_eng.pdf?dl=0) . More frequently  Less frequently  To what extent do you agree with the frequency **Duration of contractions** is being recorded? [Click heretoseesection5](https://www.dropbox.com/s/ppas54rlqswvam0/PDF_Section%205_eng.pdf?dl=0) .  Strongly disagree Disagree Agree Strongly agree |

| INTERNATIONAL CONSULTATION ON THE NEW WHO PARTOGRAPH |
| --- |
| **Section 5: Labour progress** |
| How often would you record **Duration of contractions**? [Click heretosee section5](https://www.dropbox.com/s/ppas54rlqswvam0/PDF_Section%205_eng.pdf?dl=0) . More frequently  Less frequently  To what extent do you agree with the frequency **Cervix** is being recorded? [Click heretoseesection5](https://www.dropbox.com/s/ppas54rlqswvam0/PDF_Section%205_eng.pdf?dl=0) .  Strongly disagree Disagree Agree Strongly agree |

| INTERNATIONAL CONSULTATION ON THE NEW WHO PARTOGRAPH | |
| --- | --- |
| **Section 5: Labour progress** | |
| How often would you record **Cervix**? [Click heretoseesection5](https://www.dropbox.com/s/ppas54rlqswvam0/PDF_Section%205_eng.pdf?dl=0) . More frequently  Less frequently  To what extent do you agree with the frequency **Descent** is being recorded? [Click heretoseesec](https://www.dropbox.com/s/ppas54rlqswvam0/PDF_Section%205_eng.pdf?dl=0)  Strongly disagree Disagree Agree | [tion5](https://www.dropbox.com/s/ppas54rlqswvam0/PDF_Section%205_eng.pdf?dl=0) .  Strongly agree |

| INTERNATIONAL CONSULTATION ON THE NEW WHO PARTOGRAPH |
| --- |
| **Section 5: Labour progress** |
| How often would you record **Descent**? [Click heretoseesection5](https://www.dropbox.com/s/ppas54rlqswvam0/PDF_Section%205_eng.pdf?dl=0) . More frequently  Less frequently  Is there any additional variable that you would consider as very relevant (rated 7 to 9 on a 9-point scale) for inclusion in this section? No  Yes (please specify)  Please use the box below to provide any additional comments or suggestions you may have on this section. |

| INTERNATIONAL CONSULTATION ON THE NEW WHO PARTOGRAPH |
| --- |
| **Section 6: Medication** |
| 1. **About this section: This section aims to encourage the consistent recording of all types of medication used during labour.**   [**Click here to see section 6**](https://www.dropbox.com/s/wyuaryiu67tmfxq/PDF_Section%206_eng.pdf?dl=0) **on the new partograph.**   1. **Survey questions: Please rate each of the variables below with the option that best represents your opinion.**   Please rate your opinion using a 9-point scale. Note: U/L=units per litre.  Is each term easy to understand as presented How relevant is each variable for labour in this section? Consider 1=very unclear; management? Consider 1=not relevant;  9=very clear 9=extremely relevant Oxytocin (U/L, drops/min)  Medicine IV fluid  To what extent do you agree with the frequency **Oxytocin (U/L, drops/min)** is being recorded? [Click heretoseesection5](https://www.dropbox.com/s/wyuaryiu67tmfxq/PDF_Section%206_eng.pdf?dl=0) .  Strongly disagree Disagree Agree Strongly agree |

| INTERNATIONAL CONSULTATION ON THE NEW WHO PARTOGRAPH |
| --- |
| **Section 6: Medication** |
| How often would you record **Oxytocin (U/L, drops/min)** ? [Click heretoseesection5](https://www.dropbox.com/s/wyuaryiu67tmfxq/PDF_Section%206_eng.pdf?dl=0) . More frequently  Less frequently  To what extent do you agree with the frequency **Medicine (U/L, drops/min)** is being recorded? [Click heretoseesection5](https://www.dropbox.com/s/wyuaryiu67tmfxq/PDF_Section%206_eng.pdf?dl=0) .  Strongly disagree Disagree Agree Strongly agree |

| INTERNATIONAL CONSULTATION ON THE NEW WHO PARTOGRAPH | |
| --- | --- |
| **Section 6: Medication** | |
| How often would you record **Medicine**? [Click here to see section 5](https://www.dropbox.com/s/wyuaryiu67tmfxq/PDF_Section%206_eng.pdf?dl=0) . More frequently  Less frequently  To what extent do you agree with the frequency **IV fluid** is being recorded?  Click here to see section 5  Strongly disagree Disagree Agree | Strongly agree |

| INTERNATIONAL CONSULTATION ON THE NEW WHO PARTOGRAPH |
| --- |
| **Section 6: Medication** |
| How often would you record **IV fluid**? [Click heretoseesection5](https://www.dropbox.com/s/wyuaryiu67tmfxq/PDF_Section%206_eng.pdf?dl=0) . More frequently  Less frequently  Is there any additional variable that you would consider as very relevant (rated 7 to 9 on a 9-point scale) for monitoring medication administrated during labour?  No  Yes (please specify)  Please use the box below to provide any additional comments or suggestions you may have on this section. |

| INTERNATIONAL CONSULTATION ON THE NEW WHO PARTOGRAPH |
| --- |
| **Section 7: Shared decision-making** |
| 1. **About this section: This section aims to encourage the consistent recording of all assessments and actions triggered by abnormal observations during labour.** [**Click here to see section 7**](https://www.dropbox.com/s/gsiefrgss8wtone/PDF_Section%207_eng.pdf?dl=0) **on the new partograph.** 2. **Survey questions: Please rate each of the variables below with the option that best represents your opinion.**   Please rate your opinion using a 9-point scale.  Is each term easy to understand as presented How relevant is each variable for labour in this section? Consider 1=very unclear; management? Consider 1=not relevant;  9=very clear 9=extremely relevant Assessment  Plan  To what extent do you agree with the frequency **Assessment** is being recorded?  Strongly disagree Disagree Agree Strongly agree |

| INTERNATIONAL CONSULTATION ON THE NEW WHO PARTOGRAPH | |
| --- | --- |
| **Section 7: Shared decision-making** | |
| How often would you record **Assessment**? [Click heretoseesection7](https://www.dropbox.com/s/gsiefrgss8wtone/PDF_Section%207_eng.pdf?dl=0) . More frequently  Less frequently  To what extent do you agree with the frequency **Plan** is being recorded? [Click heretoseesection](https://www.dropbox.com/s/gsiefrgss8wtone/PDF_Section%207_eng.pdf?dl=0)  Strongly disagree Disagree Agree | [7](https://www.dropbox.com/s/gsiefrgss8wtone/PDF_Section%207_eng.pdf?dl=0) .  Strongly agree |

| INTERNATIONAL CONSULTATION ON THE NEW WHO PARTOGRAPH |
| --- |
| **Section 7: Shared decision-making** |
| How often would you record **Plan**? [Click heretoseesection7](https://www.dropbox.com/s/gsiefrgss8wtone/PDF_Section%207_eng.pdf?dl=0) . More frequently  Less frequently  Is there any additional variable that you would consider as very relevant (rated 7 to 9 on a 9-point scale) for monitoring medication administrated during labour?  No  Yes (please specify)  Please use the box below to provide any additional comments or suggestions you may have on this section. |

| INTERNATIONAL CONSULTATION ON THE NEW WHO PARTOGRAPH |
| --- |
| **Section 8: Birth outcomes** |
| [About this section: This section provides minimum birth outcome data. Click here to see section 8 on the new partograph.](https://www.dropbox.com/s/e7dfreppvshh05u/PDF_Section%208_eng.pdf?dl=0)  1. **Survey questions: Please rate each of the variables below with the option that best represents your opinion.**   Please rate your opinion using a 9-point scale.  Is each term easy to understand as presented How relevant is each variable for labour in this section? Consider 1=very unclear; management? Consider 1=not relevant;  9=very clear 9=extremely relevant Mode of birth  Apgar score at 5 minutes Blood loss  Neonatal status Birthweight  How relevant would you rate having this whole section being included in the partograph? Please rate your opinion using a 9-point scale, where 1 represents not relevant and 9 extremely relevant.  1 2 3 4 5 6 7 8 9  To what extent do you agree with recording **Mode of birth** in an open text?  Strongly disagree Disagree Agree Strongly agree |

INTERNATIONAL CONSULTATION ON THE NEW WHO PARTOGRAPH

**Section 8: Birth outcomes**

[Would you rather register data using a checkbox or coding for **Vaginal (Spontaneous/Assisted)/Cesarean**? Click here to see section 8.](https://www.dropbox.com/s/e7dfreppvshh05u/PDF_Section%208_eng.pdf?dl=0)

Yes

No (please specify)

To what extent do you agree with recording **Blood loss** in an open text? Click heretoseesection8 .

Strongly disagree Disagree Agree

Strongly agree

| INTERNATIONAL CONSULTATION ON THE NEW WHO PARTOGRAPH |
| --- |
| **Section 8: Birth outcomes** |
| Would you rather register data using a checkbox with **<500ml/≥500m**l? [Click heretoseesection8](https://www.dropbox.com/s/e7dfreppvshh05u/PDF_Section%208_eng.pdf?dl=0) . Yes  No (please specify)  To what extent do you agree with recording **Neonatal status** in an open text? [Click heretoseesection8](https://www.dropbox.com/s/e7dfreppvshh05u/PDF_Section%208_eng.pdf?dl=0) .  Strongly disagree Disagree Agree Strongly agree |

| INTERNATIONAL CONSULTATION ON THE NEW WHO PARTOGRAPH |
| --- |
| **Section 8: Birth outcomes** |
| Would you rather register neonatal status using a checkbox or codes for **alive/dead**? [Click heretoseesection8](https://www.dropbox.com/s/e7dfreppvshh05u/PDF_Section%208_eng.pdf?dl=0) . Yes  No (please specify)  Is there any additional labour outcome that you would consider as very relevant (rated 7 to 9 on a 9-point scale ) to be reported in the partograph?  Yes No |

| INTERNATIONAL CONSULTATION ON THE NEW WHO PARTOGRAPH |
| --- |
| **Section 8: Birth outcomes** |
| Which of the following variables would you add to this section? You can select one or more options. [Click heretoseesection8](https://www.dropbox.com/s/e7dfreppvshh05u/PDF_Section%208_eng.pdf?dl=0) .  Timing of uterotonics Duration of third stage Placenta weight  Initiation of breastfeeding Stilbirth  Other (please specify)  Please use the box below to provide any additional comments or suggestions you may have on this section. |

| INTERNATIONAL CONSULTATION ON THE NEW WHO PARTOGRAPH |
| --- |
| **General evaluation of the new WHO partograph** |
| To what extent do you agree with the following statements about the new partograph? [Click heretoseethepartograph](https://www.dropbox.com/s/pm8cwp48itjnizw/PDF_Full%20Tool_eng.pdf?dl=0) .  Strongly disagree Disagree Agree Strongly agree It will facilitate clinical  decision-making  The reference thresholds will trigger further assessment and necessary action  It will facilitate implementation of respectful maternity care policy  Contains all relevant variables  It will be easy to complete  Instructions are clear Abbreviations are clear  The sections are logically organized  24-hour time formal is very appropriate  Enables providers identification  Comments |

The new tool is labelled as “WHO labour care guide”, in your opinion, what would be the most appropriate name for the new WHO partograph?

WHO labour care guide WHO intrapartum care tool WHO partograph

WHO labour monitoring tool Other (please specify)

| INTERNATIONAL CONSULTATION ON THE NEW WHO PARTOGRAPH |
| --- |
| **General information** |
| Country where you work?    Other (please specify)  Gender  Female Male  Your age  Under 30  30-44  45-60  >60  Which of these job designations best describes you? Nurse-midwife  Midwife  Obstetrician (Consultant) Specialist obstetric registrar General practitioner  Other (please specify)  Time since qualification (in years) <5  5-20  >20 |

Last time you worked in a labour ward (in years)

<5

5-20

>20

I have never worked in labour wards

Last time you used a partograph (in years) <5

5-20

>20

I have never used a partograph

| INTERNATIONAL CONSULTATION ON THE NEW WHO PARTOGRAPH |
| --- |
|  |
| Thank you for taking the time to complete this survey. We truly value the information you have provided. Many thanks.  **Participation certificate**  If you wish to obtain a certificate of your participation in this study, please [click here](https://es.surveymonkey.com/r/your_participation).  **Disclaimer**  The final version of the partogram is not yet finished. This version should not be used to assist women in labor. |
