# Supplementary material for: The development of the WHO Labour Care Guide: an international survey of maternity care providers
Source: Reprod Health. 2021 Mar 22;18:66. doi: 10.1186/s12978-021-01074-2 (PMC7986022; doi:10.1186/s12978-021-01074-2)
Supplement: Supplementary file 3 — Additional file 3. Methodological details. [file 12978_2021_1074_MOESM3_ESM.docx]

# Additional file 3: methodological details

***Analysis of Disagreements***

For the measurement of disagreement, the RAND DI (Disagreement Index) was applied. DI is composed of two major components: (i) the inter-percentile range (IPR) and (ii) the inter-percentile range adjusted for symmetry (IPRAS). The IPRAS was calculated with the following equation: IPRAS = IPRr + (AI * CFA) , where IPRr is the inter-percentile range required for disagreement when there is perfect symmetry (constant of 2.4); the AI is the asymmetry index, which is the distance between the centre point of the IPR and the centre point of a 9-point scale; and CFA is the correction factor for asymmetry (constant set at 1.5). The RAND DI was calculated with the following equation:

DI = IPR/IPRAS.

If the DI for an item was => 1.0, it indicated disagreement on the ratings. If the DI was < 1.0 (including negative values) it indicated agreement.
