# Supplementary material for: The development of the WHO Labour Care Guide: an international survey of maternity care providers
Source: Reprod Health. 2021 Mar 22;18:66. doi: 10.1186/s12978-021-01074-2 (PMC7986022; doi:10.1186/s12978-021-01074-2)
Supplement: Supplementary file 4 — Additional file 4. Findings from open-ended questions. [file 12978_2021_1074_MOESM4_ESM.docx]

# Additional file 4. Findings from open-ended questions

| General comments:  Respondents expressed acceptability of the tool and assured implementation feasibility.  Very few respondents expressed their preference of previous partograph for specific sections.  Design and display suggestions: add more room for written individualized comments and for provider´s name, highlight alert column, and provide better guidance on how frequently to record some parameters. | |
| --- | --- |
| *Section 1 - Woman identification and labour admission characteristics*  Labour Onset: include a definition and specify response options.  Active labour diagnosis: difficulties registering the start of active phase if the patient is admitted late.  Risk factor: include a definition.  Include maternal and foetal clinical variables and administrative information that might help to follow-up the patient. | *Section 4: Care of the mother*  Pulse: change the reference **threshold** of the alert to 60-110.  Blood pressure: merge systolic and diastolic blood pressure.  Urine: lack of clarity on how to record it. Include “P” and “A” as abbreviations. Add guide about frequency of recording (“check urine every 4-12 hours”). |
| *Section 2: Supportive care*  Companionship: replace the term according to the country “Conjoint”.  Coping: considered as a soft assessment based on subjective observation. Problems with the translation into Spanish and French.  Pain relief: suggestion to split it into pharmacological and not pharmacological. Replace with "Effective pain relief".  Posture: Problems with recording options (i.e. women is walking). Acronyms missing in the abbreviations section. | *Section 5: Labour progress*  Cervix plot: lack of clear definition, problems to understand how to record and alerts meaning  Some participants would start recording cervix dilatation at an earlier stage (before 5cm) and expressed their preference for the previous partograph display.  Descent Plot 0: Lack of clear definition; problems to understand how to record and alerts meaning. Suggestion using DeLee -3, -2, -1, 0, +1, +2, +3 for registering descent or “planos de Hodge “(Spanish).  Duration of contractions: Lack of clear definition of variable and unit of measure. |
| *Section 3: Care of the baby*  FHR Deceleration: confusion about how to record it. Suggestions to register decelerations with a different nomenclature (e.g. DIPS in Spanish).  Caput and Moulding: subjective and culturally bound variables. Symbols “+++” are not clear. Suggestion using numbers or yes/no options.  Time frames: problems regarding how frequent to record this section and to do the examination  Add place to register FHR variability and fetal movements. | *Section 6: Medication*  Medication: concerns on how to record drugs (by name, by group i.e. antibiotics, analgesia, anti-hypertensive).  IV Fluid: not clear if fluids used to dilute medication should be recorded  Oxytocin: to add abbreviations  To add place to register: Use of Oxygen |
| *Section 7: Shared decision-making*  Assessment and Plan: could not find different definition among these variables | *Section 8: Birth Outcomes*  Apgar score at 5 min: suggestion to record Apgar 1 / 5 /10 min.  To add place to register: any neonatal abnormality, newborn sex, interventions at 3rd stage of labour. |
